# Lithium concentration in tap water, bottled mineral water, and Danube River water in Hungary

**DOI:** 10.1038/s41598-023-38864-6

**Published:** 2023-08-02

**Authors:** Péter Dobosy, Ádám Illés, Anett Endrédi, Gyula Záray

**Affiliations:** 1 Institute of Aquatic Ecology, Centre for Ecological Research, Karolina út 29, Budapest, 1113 Hungary; 2 National Laboratory for Water Science and Water Security, Institute of Aquatic Ecology, Centre for Ecological Research, Karolina út 29, Budapest, 1113 Hungary; 3 Institute of Chemistry, Eötvös Loránd University, Pázmány Péter sétány 1/A, Budapest, 1117 Hungary

**Keywords:** Environmental chemistry, Environmental impact

## Abstract

Due to increased manufacture and recycling of lithium batteries across the world, we may anticipate a rise in lithium pollution in the aquatic environment and drinking water reservoirs. In order to investigate the current status regarding the lithium content in Hungarian tap waters, samples were collected from the public drinking water supply systems of 19 county seats in Hungary during seasonally selected times. Depending on the water sources, such as bank-filtrated river water, surface water from open reservoirs, and groundwater, the lithium concentrations varied between 0.90–4.23, 2.12–11.7 and 1.11–31.4 µg/L, respectively, while the median values were 3.52, 5.02 and 8.55 µg/L, respectively. The lithium concentration in the bottled Hungarian mineral waters was also determined since the daily intake of lithium can be influenced by the consumption of mineral waters. The concentrations ranged from 4.2 to 209 µg/L, while the median value was only 17.8 µg/L. Additionally, a correlation was only found between lithium and potassium concentrations. The lithium concentration was also assessed at ten sampling locations in the Hungarian segment of the Danube River since the Danube water is also a water source for additional drinking water utilities using bank filtration technology. The mean and median lithium concentrations were 2.78 and 2.64 µg/L, respectively.

## Introduction

Due to increasing lithium (Li) battery manufacturing and application, as well as the recycling of used batteries, it is necessary to estimate rising Li levels in the environment^1–4^. The surface waters are particularly at risk since the Li ions cannot be eliminated using conventional biological treatment methods in wastewater treatment plants (WWTPs). A recent study found that the concentration of lithium in the influent and effluent of a secondary WWTP in Seoul were essentially the same ranging from 0.69 to 8.2 µg/L^3^. As a result, it is anticipated that the high mobility lithium ions will pass through the bank filtration systems which based also on microbiological degradation processes of organic contaminants, furthermore adsorption and complex or chelate formation of metal ions with the functional groups of biofilms^5^. However, due to their low positive charge (+ 1) and relatively large ionic radii, the alkali metal cations have only a week tendency to form complexes with simple Lewis basis. Therefore, increasing lithium concentration in rivers leads to increasing lithium concentration in bank filtrated water and at the end in the drinking water.

Li has not yet been categorized as an essential trace element for humans. Its recommended dietary allowance for adults with a body weight of 70 kg is 1 mg/day^6^. The primary dietary sources of Li are cereals, potatoes, tomatoes, cabbage, and mineral waters from specific locations^7,8^. According to estimates, cereal grains and vegetables can provide between 66 and 90% of the daily Li consumed^9^. The remainder is supplied by animal-derived foods, drinking water, and beverages. Due to its normothymic effects, Li in the form of carbonate has become one of the most commonly utilized medications in psychiatric treatment. Since 1949, it has been used for treating bipolar disorder^10^, and it is still recommended for treating acute mania and manic episodes^11,12^. The typical therapeutic oral dosages of lithium carbonate per day vary from 600 to 1200 mg^13^. Using highly bioavailable orotate chelate, a low-dose Li therapy was also developed^14^. Marshall et al.^15^ provided an overview of the biochemical processes and effects generated by Li ions and Li chelates in humans.

A number of studies have investigated the relationship between Li concentration in drinking water and the risk of suicide, homicide, and arrest rate for drug use^16–35^. Most of these studies indicate a link between higher Li concentrations in drinking water and a lower risk of suicide. As a result of these medical-biological investigations, a lot of analytical data on the Li concentration of drinking waters derived from diverse water sources around the world is now available. Table S1 contains a list of the published Li-concentrations. These findings demonstrate that, notwithstanding the lack of knowledge on the sources of these drinking waters, Li concentrations measured in drinking waters in various countries varied by three orders of magnitude, and the published mean concentrations ranged from 0.48 to 56 µg/L.

Several other studies^36,37^ examined the Li content of groundwater (bank-filtrated and karst waters) and surface waters (rivers, lakes, open reservoirs) used to supply drinking water in the United States. Li concentrations in groundwater ranged between 1 and 396 µg/L (median 8.1 µg/L) for public supply wells and 1–1700 µg/L (median 6 µg/L) for domestic supply wells nationwide. Sharma et al.^37^ investigated the presence of Li in water sources (ground and surface water) across the United States in 21 drinking water facilities. In groundwater, Li concentrations ranged from 0.9 to 161 µg/L (median 13.9 µg/L), and in surface water, they ranged from 0.5 to 130 µg/L (median 3.9 µg/L). Li levels were higher than the non-regulatory Health-Based Screening Level (HBSL) of 10 µg/L in 56% of the groundwater and 13% of the surface water^36^. The authors also discovered a strong correlation between Li and sodium (Na) concentrations. Additionally, it was observed that the Li concentration in source water and treated drinking water were remarkably similar.

For the purpose of calculating the dietary intake of Li, bottled mineral waters should also be taken into account due to the rising consumption of these waters and their high mineral content. In 2019, Italy was the largest consumer of mineral waters in the European Union, consuming 200 L per capita per year, while Hungary consumed 139 L per capita yearly or 380 mL per day^38^, placing it fourth overall. Since the chemical composition and Li content of natural mineral waters greatly depend on the geochemical composition of lithological strata in aquifers, it is expected that the Li concentration of mineral waters from different countries will vary widely. In Germany, Seidel et al.^39^ measured the concentration of Li in 360 mineral waters and 21 medicinal waters. The mean and median Li concentrations of all the analyzed mineral and medicinal waters were 107 µg/L and 31.2 µg/L, respectively. The majority of the waters that were analyzed had Li concentrations below 50 µg/L. The Li concentration showed significant correlations with the Na and potassium (K) concentrations. It should be noted that eight years prior, Birke et al.^40^ had made comparable observations while researching several German mineral waters, with the median Li concentration being 29 µg/L. In Portugal, Neves et al.^41^ investigated 18 brands of mineral waters and separated them into two groups with relatively low (less than 11 µg/L) and significantly higher (more than 100 µg/L) Li concentrations. The highest Li concentration (1500 µg/L) was measured in a highly mineralized Na–HCO_3_-type water.

River waters comprise the third group of water matrices and are significant recipients of treated industrial wastewater. Due to the high water consumption of the hydrothermal recycling process for spent Li batteries, it is anticipated that Li concentrations would rise, particularly in rivers where treated industrial wastewater is discharged. It is likely that the bank-filtration technology used for producing drinking water along the larger rivers may not be suitable for removing this target element. In order to evaluate the potential risk and effect of Li contamination on the aquatic life of rivers, it is essential to first determine the characteristic Li concentration values in the river waters. Depending on the geochemical composition of the bedrock and weathering processes in the catchment area, the meteorological conditions, and the natural and anthropogenic contaminants of tributaries, the dissolved Li concentration in rivers varies widely (0.36–454 µg/L)^42–47^.

The aim of this study was to determine the Li concentration in tap waters of 19 Hungarian county seats (drinking water was derived from bank-filtrated water, surface water from open reservoirs, or groundwater) as well as in bottled Hungarian mineral waters. These data will assist us in calculating the daily intake of Li from tap and mineral waters that contain Li in a highly bioavailable form. The Li concentrations were also measured at ten sampling sites along the Hungarian segment of the Danube River to determine the typical input values for bank-filtration technology in 2021 and 2022. These concentrations will serve as a reference value (background level) to evaluate the anticipated effects of anthropogenic Li on the aquatic environment over the ensuing years.

## Results and discussion

### Tap water

**Figure 1:**
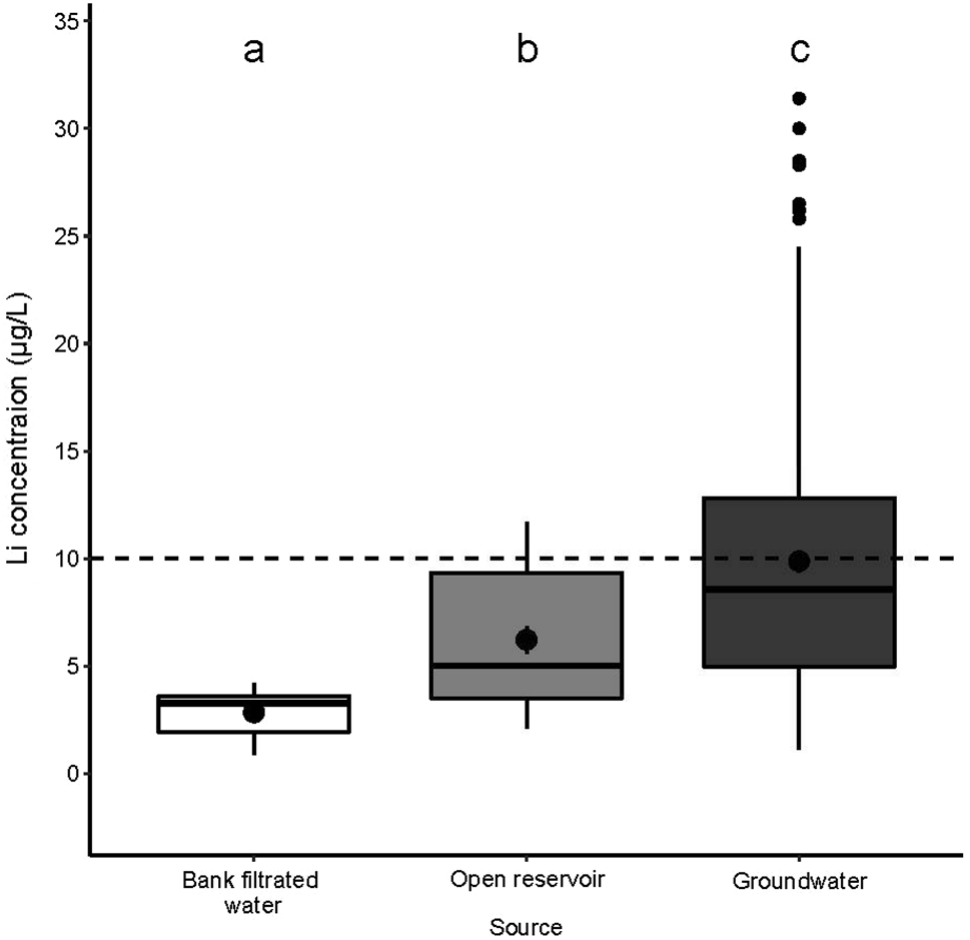
Lithium distribution in tap water samples collected from public drinking water network of 19 county seats grouped by their water source (bank-filtrated waters, open reservoirs, or groundwaters). Bold horizontal lines within the boxes show the median (middle value) of the dataset, the lower edges of the boxes represent the first quartile, while the upper edges show the third. Points and small vertical lines within the boxes show the mean values and their standard errors. Alphabets above the boxes present the results of statistical analysis (linear model). Different characters show significant (*p* < 0.05) differences in mean values. The dashed line indicates the 10 µg/L non-regulatory Health-Based Screening Level.

Table 1 lists the minimum and maximum concentrations, as well as the mean and median values of Li, and Table S2 displays the physico-chemical parameters of the samples. The Li concentration of tap water is influenced by the water source used to produce the drinking water, as can be inferred from Fig. 1. The median concentration increased in the following order: bank-filtrated water < surface water from open reservoir < groundwater, including karst water. However, it is important to note that in the case of all three water sources, the median concentrations remained below the non-regulatory HBSL of 10 µg/L. The highest mean concentration range for Li was measured in tap waters derived from groundwater (1.11–31.4 µg/L) originating from coarser sandy and gravel layers of clastic basin deposits, or from greater depths where sandstone can be found instead of the loose sandy layers. These aquifers can be found in over three-quarters of the country’s land, ensuring the possibility of local drinking water production everywhere. The highest Li concentration (31.4 µg/L) was detected in karst water-based tap water, which is present in about half of Hungary’s hilly areas, which make up one-fifth of its total land area^48^. The mean Li concentrations in tap water derived from open reservoirs ranged between 3.5 and 8.9 µg/L. Since rainwater carries Li only on low concentrations of 0.1–1.0 µg/L^49^, in open reservoirs the elemental content of water is mostly influenced by atmospheric deposition, mountains streams, and the dissolution of minerals in the bottom layer. The lowest Li concentrations were detected in tap water derived from bank-filtrated waters. The mean concentrations of Li changed in the range of 1.5–3.6 µg/L. It should be noted that bank-filtration technology accounts for 44% of Hungarian drinking water production, including the water supply to the nation’s capital.

**Table 1:** The minimum and maximum, as well as the mean and median concentrations of lithium in tap waters of 19 county seats indicating the water sources for the production of drinking water at different locations.

Location	Water source	Min	Max	Mean (± SD)	Median
Budapest	Bank-filtrated water	2.52	4.20	3.40 (0.45)	3.55
Győr	Bank-filtrated water	2.95	4.23	3.60 (0.33)	3.59
Szekszárd	Bank-filtrated water	0.90	2.27	1.56 (0.42)	1.57
Salgótarján	Open reservoir	5.05	11.7	8.89 (2.18)	9.45
Szolnok	Open reservoir	2.12	4.99	3.56 (0.81)	3.51
Miskolc	Groundwater	8.04	14.3	11.1 (2.0)	10.9
Pécs	Groundwater	8.99	14.8	10.7 (1.8)	10.5
Székesfehérvár	Groundwater	13.9	24.5	19.8 (3.2)	20.5
Tatabánya	Groundwater	3.01	8.80	5.92 (1.96)	6.02
Veszprém	Groundwater	1.11	4.20	2.64 (1.03)	2.57
Békéscsaba	Groundwater	1.52	3.00	2.32 (0.47)	2.31
Debrecen	Groundwater	4.44	8.54	6.13 (1.40)	5.88
Eger	Groundwater	20.1	31.39	25.7 (3.5)	26.0
Kaposvár	Groundwater	9.84	14.93	12.4 (1.6)	12.5
Kecskemét	Groundwater	4.01	6.80	5.41 (0.90)	5.46
Nyíregyháza	Groundwater	4.95	10.5	7.78 (1.63)	8.06
Szeged	Groundwater	3.39	8.68	5.56 (1.68)	5.00
Szombathely	Groundwater	5.26	11.6	8.44 (1.82)	8.70
Zalagerszeg	Groundwater	11.1	17.9	14.3 (2.1)	14.3

**Figure 2:**
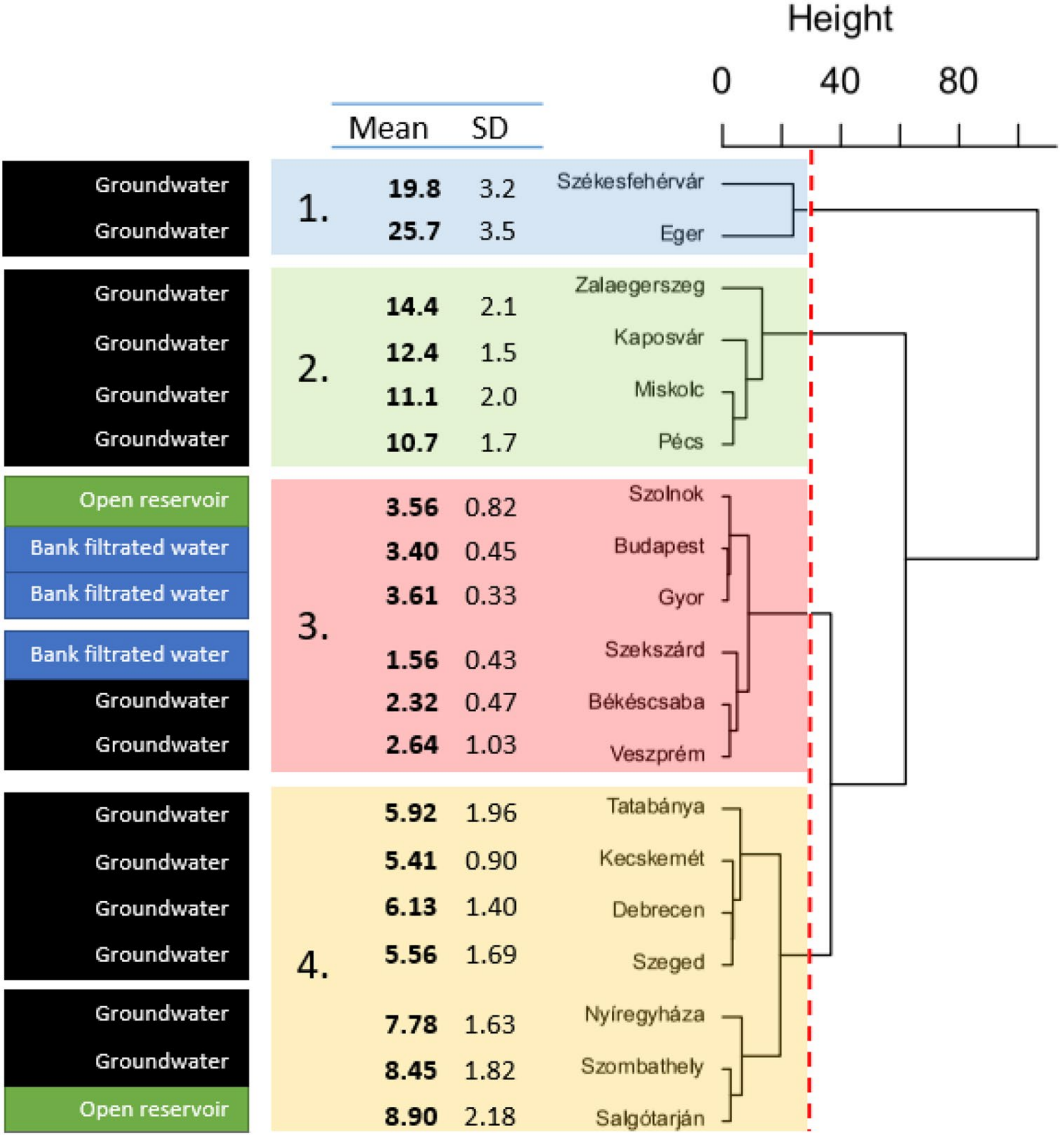
Hierarchical clustering of the 19 cities based on the lithium content of their tap water. The clustering is based on Euclidean distances, and four major groups were formed.

Hierarchical clustering was used to group the cities based on the amount of Li in their tap water (Fig. 2). Based on the structure of the dendrogram and the similarities of the cities’ mean Li values we separated four different groups. The first group includes the two cities with the highest mean Li values (25.7 and 19.8 µg/L) in tap water, while the second group includes four cities with a mean Li concentration between 10.75 and 14.35 µg/L. The third group featured the cities with the lowest mean Li concentration (between 1.56 and 3.61 µg/L) in their tap water, while the fourth group included cities with Li concentration of 5.81–8.90 µg/L in their tap water (Fig. 2). We investigated if the type of water source (i.e., bank-filtrated, open reservoir, or groundwater) may account for the clustering and, thereby, the similarity in Li concentrations in the different cities. Although all the three cities (Budapest, Győr, and Szekszárd) which received bank-filtrated water as tap water were grouped together in the third group, tap waters originating from open reservoirs or groundwaters were mixed in the other groups. Additionally, because cities from different geographical regions (groups (see their locations within the country on Fig. 1) were mixed in all groups, thus, the majority of the variance between the cities cannot be explained by only the type of water source or the spatial distances between the different cities.

Li concentrations in tap waters are the subject of numerous studies conducted worldwide, and the concentration values fluctuate in a very wide range. The majority of studies are from Japan, where Li concentrations ranged from < 1 to 59 µg/L^16,22,26^, and were in some cases very similar to our results. However, lower concentrations (0.01–2.10 µg/L)^50^ and higher concentrations (0–130 µg/L) were also measured^23,33^. Li concentration in drinking water in England fluctuated between < 1 and 1300 µg/L^51^, while in South Korea the concentrations remained below 1 µg/L^3^. Other studies reported that the Li concentrations in Texas, Greece, and Portugal varied in the following concentration ranges: 2.80–539 µg/L^21,29^, 0.10–121 µg/L^20^, and 0.10–191^31^, respectively. Results from Denmark^52,53^, Italy^25^, Lithuania^27^, and the USA (Alabama)^32^, where Li amounted to 0.60–30.7, 2.0–27, 0.11–60.8, and 0.48–39 µg/L, respectively, appeared to be quite comparable to those from the public network water in Hungary (0.40–32.9 µg/L).

### Mineral water

**Figure 3:**
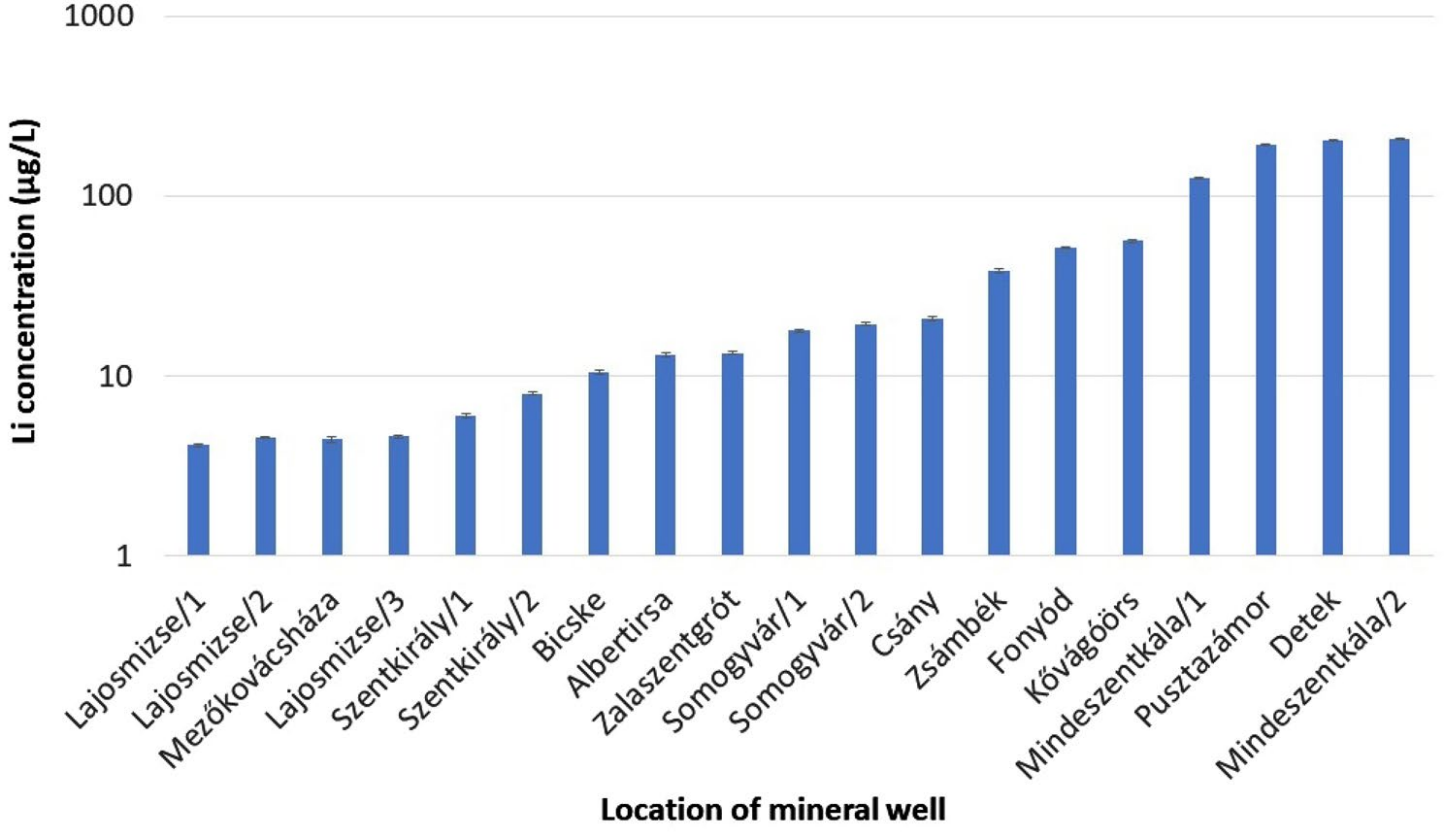
Lithium concentration in bottled Hungarian mineral waters.

**Figure 4:**
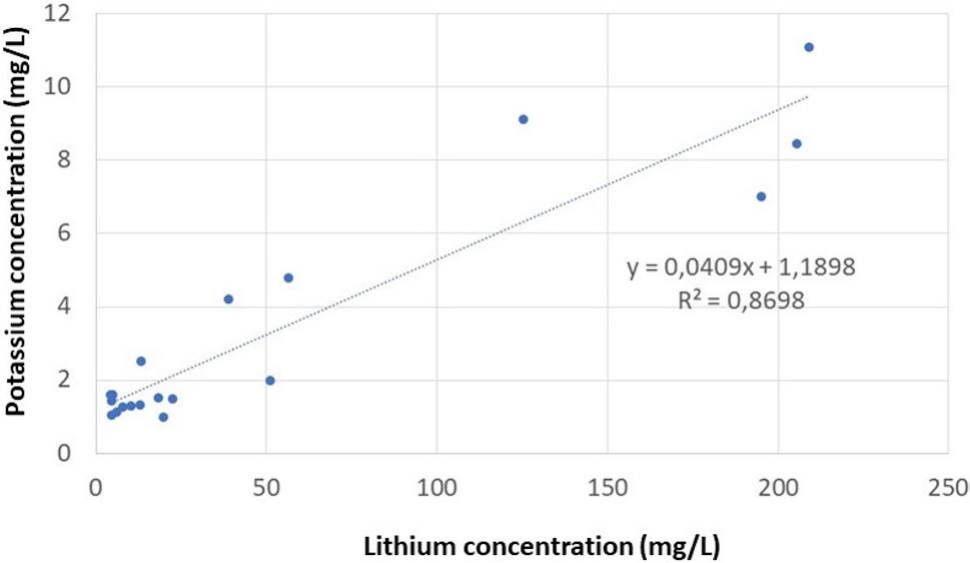
Correlation between lithium concentration and potassium concentration in mineral waters.

The concentration of cations and anions in mineral waters is determined by the chemical composition of the aquifer or underground reservoir. Consequently, the concentration of cations and anions varies in a wide range. Mineral waters are classified into different water types based on their dominant constituents. In Hungary, the Na/HCO_3_ and Ca–Mg/HCO_3_ water types are predominant, and the total dissolved mineral content varies between 360 and 1600 mg/L. The following parameters are typically listed on the bottles to describe the mineral waters for the consumer: the total amount of dissolved minerals, the concentration of four cations (Ca^2+^, Mg^2+^, Na^+^, K^+^), and four anions (Cl^−^, SO_4_^2−^, NO_3_^−^, HCO_3_^−^). These chemical parameters, together with the Li concentration, pH, and electric conductivity, were determined during our investigation and are listed in Table S3. It should be underlined that in the settlements Lajosmizse, Szentkirály, and Mindszentkála, there were more wells with varying depths and corresponding water reservoirs positioned between several aquitard layers. Based on the measured Li concentrations, three categories of mineral waters with Li contents of low (5–10 µg/L), medium (10–100 µg/L), and relatively high (100–200 µg/L) can be distinguished (Fig. 3). The mean and median Li concentrations were 52.9 and 17.9 µg/L, respectively. Comparing our findings to those of Seidel’s group from Germany revealed that their median value (31.2 µg/L) is nearly twice as high as ours, but they also analyzed medicinal waters with extremely high mineral content in addition to mineral waters. Their observation that Li concentrations are typically below 50 µg/L in the majority of analyzed waters is consistent with our findings. They also discovered significant correlations between the concentrations of Li and Na (R^2^ = 0.656) as well as Li and K (R^2^ = 0.513). However, in our mineral water samples, a correlation was observed only between the Li and K concentrations (R^2^ = 0.867) (Fig. 4). In a study conducted in Japan, Li concentrations in domestic and international mineral waters were compared, and it was found that the Li content in Japanese mineral waters was one order of magnitude lower (mean: 2.90 µg/L) than that in mineral waters from other countries (mean: 57.1 µg/L)^23^. In Portugal, 18 different mineral water brands were examined, and based on the measured Li concentrations, the brands were divided into two groups: those with low (< 11 µg/L) and high (> 100 µg/L) Li concentrations^41^.

### Danube River

**Figure 5:**
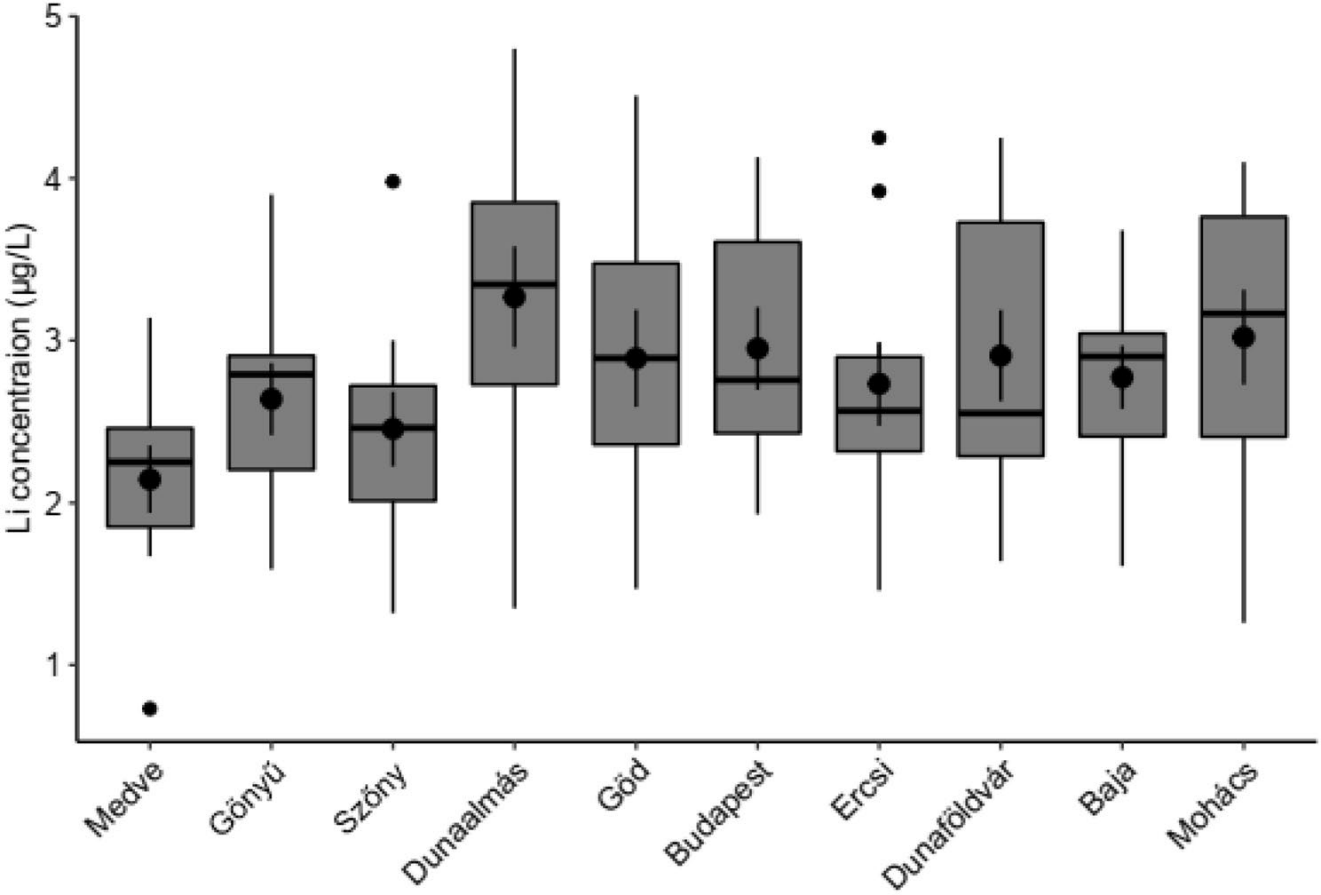
Mean + SE (dot and vertical lines within the boxes) and median (horizontal bold lines within the boxes) lithium concentrations determined in the Hungarian segment of the Danube at ten sampling sites during the time period Sept. 2021–Sept. 2022. Sampling locations are arranged from left to right following the direction of the river’s flow.

Table S4 displays the Li concentrations as well as the other chemical parameters of the Danube River measured at ten sampling locations along the 417 km Hungarian segment. The mean and median Li values are presented in Fig. 5 and Fig. S1. The results indicate that the Li concentration in the river varied within a relatively narrow range of 0.73–4.80 µg/L. Based on the data illustrated in Fig. 5 and the statistical analysis, the Li concentration in the Danube River reached its peak in Dunaalmás; it then continued to be significantly higher (*p* < 0.05, paired *t*-test) at all other sampling locations. In the upper zone (at Medve, Gönyű, and Szőny), the average Li concentration ranged between 2.15 and 2.64 µg/L, whereas at Dunaalmás, it increased to an average of 3.27 µg/L. The subsequent downstream sampling sites displayed median values and did not significantly differ from those collected at Dunaalmás. Li concentrations in the Danube River were comparable to those in other rivers around the world, but in some instances, significantly different concentrations were observed. Huh et al.^42^ investigated Li and its isotopes in major world rivers (Yana, Indigirka, Fraser, Columbia, Mackenzie, Ganges, Lena, Mississippi, Brahmaputra, Yangtze, Orinoco, Congo, Amazon). In these rivers, the dissolved Li concentrations fluctuated between 0.36 and 5.5 µg/L, and the discharged weighted mean value was 1.46 µg/L. In another study, low Li concentrations (0.4–0.8 µg/L) were measured in Myanmar’s Irrawaddy, Pathein, and Yangon rivers^47^. However, Qu et al.^44^ observed considerably higher Li concentrations in rivers in the “Water Tower of Asia”, where this parameter changed from 8.2 µg/L (Yellow River) to 454 µg/L (Zahija Tsangpo river). On the basis of a large spatio-temporal dataset, Boral et al.^45^ calculated the ambient background concentrations of 15 trace elements dissolved in the Ganga River. Li baseline concentrations during the monsoon (high flow) and non-monsoon (low flow) were 1.2–3.4 and 2.2.–5.4 µg/L, respectively. In Europe, Li concentrations were close to 2 µg/L in the upper sections of the Danube and Rhine^46^, whereas in the Marne and Seine rivers close to Paris, the concentration of this target element fluctuated between 3 and 6 µg/L throughout the year and reached a maximum value in November^43^. Similar concentrations (0.2–4.0 µg/L) were reported in more pristine river water systems^42^. We can assess future changes in the water quality of different rivers using findings like the one described in this research.

## Conclusion

If we accept Schrauzer’s assertion that the dietary allowance for Li is 1 mg/day and the finding that drinking water contains Li on average at concentration of 5 µg/L, then based on the measured Li concentration in Hungary, it can be deduced that when we consume daily 2 L of drinking water, we only take one-hundreds of the daily requirements. If we also consider the mineral water consumption in Hungary, the daily intake of Li from tap water and mineral water amounts to only ~ 26 µg/capita, which is less than 3% of dietary allowance. Although the highest increment in Li concentration caused by Li-battery production and recycling of spent batteries can be expected in drinking water produced from bank filtrated Danube water, this does not result in substantial change in the daily intake of the affected population. It means the dietary allowance of Li should be covered from different plant-based foods (cereals, tomatoes, potatoes, cabbage) which contain Li in a less bioavailable chemical form^39^. It should be noted that measured Li concentrations related to the Danube water serve as background values for evaluation of the expected Li contamination of the aquatic environment.

## Material and methods

### Sampling

**Figure 6:**
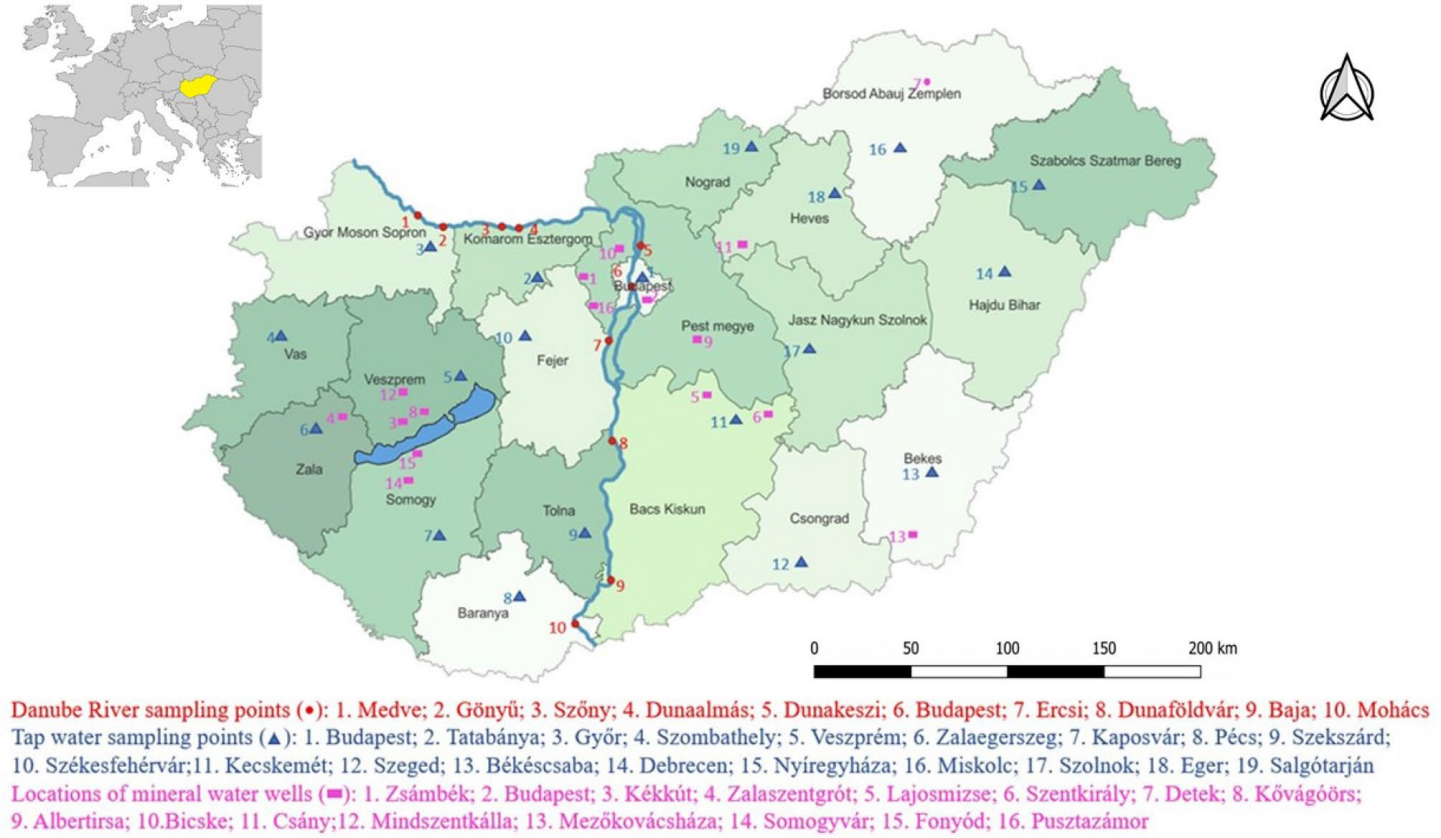
Location of county seats, mineral water wells, and sampling sites along the Danube River in Hungary.

The locations of county seats, mineral water wells, and sampling sites along the Danube River are illustrated in Fig. 6. Between September 2021 and September 2022, a monthly Danube water sampling program was conducted to collect 1 L of water from the surface layer (10–50 cm) at ten sampling sites that represented the 417 km long Hungarian segment of the Danube River. The tap water samples were collected from the public drinking water supply systems of 19 county seats in October 2021 and January, May, and August 2022. Additionally, 19 brands of bottled natural non-carbonated mineral waters from Hungary were bought at random from the market.

### Sample preparation

For the purpose of measuring the cations and anions, 500 mL of tap water samples were placed into purified glass containers without any preservation steps. Li was determined by removing the suspended particles on-site using syringe filters with 0.45 µm pore sizes, and following filtration, 10 mL of water samples were placed into polypropylene centrifuge tubes. These samples were then acidified by adding 100 µL high purity concentrated nitric acid (VWR International, Pennsylvania, USA). Cold boxes were used to transport the untreated and pre-treated water samples to the laboratory.

### Chemical analyses of waters

The pH and electric conductivity of drinking water, mineral water, and Danube River water samples were measured using a portable MultiMeter (HI98130, Hanna Instrument, USA). Alkalinity was measured by standard titrimetric method^54^. The concentration of the principal cations (Ca^2+^, Mg^2+^, Na^+^, K^+^) and anions (Cl^−^, SO_4_^2−^, NO_3_^−^) were determined in all water samples by a dual channel ion chromatograph (Dionex ICS 5000, Thermo Fischer Scientific, USA). The limit of quantification for these analytes was close to 1 mg/L. Li concentrations were quantified using an inductively coupled plasma mass spectrometer (PlasmaQuant Elite, Analytik Jena, Germany), applying ^45^Sc at a concentration of 20 µg/L as the internal standard. The limit of quantification (LOQ) for Li was 0.537 µg/L.

### Statistical analysis

Data were analyzed and visualized by R statistical software^55^. Mean Li values of the tap water samples (depicted in Fig. 1) originating from different sources (i.e., bank-filtrated, open reservoir, and groundwater) were compared in R software by the linear model (‘lm’ function of the *stats* package). Post-hoc pairwise comparisons were made by Tukey contrasts (computed by the ‘glht’ function of the *multcomp* package^56^. Li values were log-transformed before analysis to meet the assumptions of the linear model. The median and mean of the Li values measured in tap water and Danube River water samples were drawn in R using the ‘ggboxplot’ function of the *ggpubr* package^57^. Figure 2 depicts the similarities between the sampling sites and the results of hierarchical cluster analysis made by the ‘hclust’ function of the *stats* package in R software For the analysis, we used the original (not standardized) Li values that were measured in four time points (one in each seasons) for the same sampling sites (i.g., we had four different parameters for each sampling points and three sampling points in every city). In the analysis, Euclidean distances (computed by the ‘dist’ function of the *stats* package) and Ward’s minimum variance clustering method (method = “ward.D2”) were used. Based on the structure of the resulted dendrogram and the similarities of the cities’ mean Li values (depicted also on Fig. 2), we cut the tree at 30 value and divided the cities into 4 groups representing cities with similar Li concentrations (and seasonality) in their tap waters. In the case of Danube samples, Li values measured at Dunaalmás (having the highest Li concentration among the Danube River sampling points) were compared (Fig. 5) to the values at the other sites by paired t-tests (‘t.test’ function of the *stats* package), and *p*-values were adjusted by Holm’s method^58^ using the ‘p.adjust’ function of the *stats* package. Figure 6 was prepared by using QGIS 3.26 Buenos Aires softwere^59^ and the original map is freely available^60^.

## Supplementary Information

Supplementary Information.

## Data Availability

Original experimental data are available from the corresponding author upon a request.
