# Supplementary material for: Lithium concentration in tap water, bottled mineral water, and Danube River water in Hungary
Source: Sci Rep. 2023 Aug 2;13:12543. doi: 10.1038/s41598-023-38864-6 (PMC10397251; doi:10.1038/s41598-023-38864-6)
Supplement: Supplementary file 1 — Supplementary Information. [file 41598_2023_38864_MOESM1_ESM.docx]

**Supplementary Material**

**Table S1 Concentration ranges of lithium in public drinking/tap water in different countries**

| **Country** | **Li concentration range (µg/L)** | **Reference** |
| --- | --- | --- |
| Austria | 3.3-82.3 (mean: 10.0) | Helbich et al., 2015^23^ |
| Denmark | 0.6-30.7 (mean 11.6) | Kessing et al., 2017a^51^ |
| Denmark | 2-27 | Kessing et al., 2017b^52^ |
| England | <1-1300 (mean:4.2) | Kaputsa et al., 2011^50^ |
| England | <1-21 | Kabacs et al., 2011^18^ |
| Greece | 0.1-121 (mean:11.1) | Giotakos et al., 2013^19^ |
| Hungary | 0.71-89.4 | Izsák et al., 2022^33^ |
| Italy | 0.11-60.8 (mean: 5.28) | Pompili et al., 2015^24^ |
| Japan | 0.7-59 | Ohgami et al., 2009^15^ |
| Japan | <1-12.9 | Sugawara et al., 2013^21^ |
| Japan | <1-130 (mean:4.2) | Ishii et al., 2015^22^ |
| Japan | 0.1-43 | Shiotsuki et al., 2016^25^ |
| Japan | 0-130 (mean:4.2) | Kohno et al., 2020^32^ |
| Lithuania | 0.48-35.5 (mean:10.9; median:3.6) | Liaugaudaite et al., 2017^26^ |
| Lithuania | 1.0-39 (mean:11.5) | Liaugaudaite et al., 2021^57^ |
| Macedonia | 0.1-5.2 | Bauer et al., 2014^58^ |
| Portugal | 0.1-191 (mean:10.88) | Oliveira et al., 2019^30^ |
| South Korea | 0.3-0.8 | Choi et al., 2019^3^ |
| USA | <1-40 (mean:27.4) | Parker et al., 2018^29^ |
| USA (Alabama) | 0.4-32.9 | Palmer et al., 2019^31^ |
| USA (Texas) | 2.8-219 | Blüml et al., 2013^20^ |
| USA (Texas) | 3-539 (mean:56; median:40) | Fajardo et al., 2018^28^ |

**Table S2. Mean concentrations of selected cations and anions as well as pH and conductivity values of tap water samples collected seasonally from the public networks of 19 county seats (n=12)**

| **Source** | **Sampling site** | **pH** | **Electric conductivity**  **(µS/cm)** | **Na^+^**  **(mg/L)** | **Mg^2+^**  **(mg/L)** | **K^+^**  **(mg/L)** | **Ca^2+^**  **(mg/L)** | **Cl^-^**  **(mg/L)** | **SO_4_^2-^**  **(mg/L)** | **NO_3_^-^**  **(mg/L)** | **HCO_3_^-^**  **(mg/L)** |
| --- | --- | --- | --- | --- | --- | --- | --- | --- | --- | --- | --- |
| Bank filtreted water | Budapest | 7.94 ± 0.39 | 463 ± 25 | 14 ± 2 | 15 ± 1 | 2 ± 0.9 | 62 ± 6 | 20 ± 4 | 33 ± 5 | 4 ± 3 | 246 ± 20 |
| Bank filtreted water | Győr | 7.83 ± 0.14 | 542 ± 23 | 21 ± 1 | 20 ± 1 | 1 ± 0.4 | 64 ± 4 | 15 ± 3 | 41 ± 4 | 2 ± 0.6 | 295 ± 20 |
| Bank filtreted water | Szekszárd | 7.75 ± 0.26 | 646 ± 36 | 78 ±73 | 13 ± 8 | 1 ± 0.6 | 64 ± 36 | 19 ± 3 | 46 ± 5 | 2 ± 0.5 | 388 ± 51 |
| Open reservoir | Salgótarján | 7.80 ± 0.27 | 447 ± 70 | 27 ± 9 | 12 ± 4 | 4 ± 1.1 | 41 ± 9 | 26 ±5 | 65 ± 13 | 4 ± 1 | 148 ± 39 |
| Open reservoir | Szolnok | 7.94 ± 0.33 | 388 ± 124 | 21 ± 12 | 7 ± 1 | 2 ± 0.5 | 40 ± 8 | 30 ± 18 | 25 ± 4 | 4 ± 1 | 144 ± 35 |
| Groundwater | Miskolc | 7.71 ± 0.25 | 551 ± 38 | 16 ± 9 | 14 ± 7 | 3 ± 1.2 | 69 ± 11 | 25 ± 10 | 42 ± 16 | 8 ± 1 | 235 ± 46 |
| Groundwater | Pécs | 7.74 ± 0.24 | 629 ± 26 | 23 ± 7 | 30 ± 3 | 1 ± 0.5 | 79 ± 14 | 11 ± 2 | 19 ± 4 | 7 ± 3 | 428 ± 16 |
| Groundwater | Székesfehérvár | 7.85 ± 0.28 | 774 ± 111 | 27 ± 17 | 42 ± 4 | 2 ± 0.3 | 95 ± 25 | 13 ± 6 | 54 ± 13 | 10 ± 5 | 501 ± 92 |
| Groundwater | Tatabánya | 7.66 ± 0.29 | 611 ± 170 | 5 ± 1 | 46 ± 1 | 2 ± 0.5 | 88 ± 4 | 3 ± 1 | 46 ± 4 | 2 ± 0.5 | 469 ± 23 |
| Groundwater | Veszprém | 7.65 ± 0.19 | 664 ± 43 | 4 ± 1 | 40 ± 3 | 2 ± 0.5 | 67 ± 5 | 6 ± 2 | 17 ± 3 | 24 ± 5 | 376 ± 16 |
| Groundwater | Békéscsaba | 8.00 ± 0.16 | 359 ± 37 | 64 ± 7 | 4 ± 1 | 1 ± 0.5 | 24 ± 5 | 15 ± 2 | 3 ± 1 | 1 ± 0.5 | 234 ± 32 |
| Groundwater | Debrecen | 7.73 ± 0.20 | 555 ± 65 | 33 ± 11 | 18 ± 3 | 1 ± 0.6 | 71 ± 9 | 16 ± 8 | 9 ± 4 | 3 ± 1 | 346 ± 52 |
| Groundwater | Eger | 7.50 ± 0.20 | 532 ± 21 | 28 ± 3 | 11 ± 1 | 4 ± 0.9 | 76 ± 9 | 11 ± 3 | 24 ± 3 | 1 ± 0.5 | 358 ± 96 |
| Groundwater | Kaposvár | 7.69 ± 0.17 | 661 ± 15 | 52 ± 6 | 27 ± 3 | 2 ± 0.5 | 42 ± 24 | 8 ± 1 | 2 ± 1 | 1 ± 0.5 | 404 ± 64 |
| Groundwater | Kecskemét | 7.80 ± 0.19 | 551 ± 36 | 21 ± 7 | 25 ± 1 | 2 ± 0.5 | 52 ± 28 | 3 ± 1 | 2 ± 1 | 2 ± 0.6 | 335 ± 71 |
| Groundwater | Nyíregyháza | 7.66 ± 0.24 | 622 ± 26 | 50 ± 2 | 16 ± 2 | 2 ± 0.6 | 70 ± 5 | 5 ± 1 | 4 ± 1 | 3 ± 0.5 | 427 ± 26 |
| Groundwater | Szeged | 7.70 ± 0.26 | 480 ± 21 | 41 ± 8 | 21 ± 1 | 1 ± 0.5 | 39 ± 5 | 2 ± 1 | 2 ± 1 | 1 ± 0.4 | 349 ± 20 |
| Groundwater | Szombathely | 7.54 ± 0.10 | 600 ± 44 | 12 ± 1 | 24 ± 4 | 1 ± 0.2 | 63 ± 20 | 7 ± 2 | 16 ± 3 | 7 ± 1 | 335 ± 36 |
| Groundwater | Zalagerszeg | 7.81 ± 0.26 | 599 ± 109 | 89 ± 16 | 17 ± 6 | 2 ± 0.6 | 35 ± 12 | 2 ±1 | 11 ± 4 | 2 ± 0.7 | 410 ± 89 |

**Table S3. Concentrations of total dissolved solids, selected cations and anions as well as pH, and electric conductivity values of Hungarian mineral waters**

| **Well (location)** | **pH** | **Electric conductivity**  **(µS/cm)** | **Total dissolved mineral content**  **(mg/L)** | **Na^+^**  **(mg/L)** | **Mg^2+^**  **(mg/L)** | **K^+^**  **(mg/L)** | **Ca^2+^**  **(mg/L)** | **Cl^-^**  **(mg/L)** | **SO_4_^2-^**  **(mg/L)** | | **NO_3_^-^**  **(mg/L)** | **HCO_3_^-^**  **(mg/L)** | **Li**  **(µg/L)** |
| --- | --- | --- | --- | --- | --- | --- | --- | --- | --- | --- | --- | --- | --- |
| Lajosmizse/1 | 7.64 | 505 | 461 | 15 | 23 | 2 | 56 | 2 | 6 | | <1 | 323 | 4,12 |
| Lajosmizse/2 | 7.76 | 501 | 471 | 30 | 22 | 1 | 61 | 7 | 9 | | <1 | 340 | 4,58 |
| Mezőkovácsháza | 8.43 | 404 | 359 | 81 | 4 | 1 | 3 | 13 | 12 | | 2 | 116 | 4,44 |
| Lajosmizse/3 | 7.60 | 500 | 501 | 15 | 23 | 2 | 60 | 3 | 6 | | <1 | 360 | 4,61 |
| Szentkirály/1 | 7.10 | 504 | 480 | 38 | 22 | 1 | 63 | 4 | 6 | | 1 | 320 | 6,01 |
| Szentkirály/2 | 7.17 | 530 | 520 | 20 | 24 | 1 | 60 | 2 | 1 | | <1 | 378 | 7,99 |
| Bicske | 7.56 | 655 | 644 | 2 | 42 | 1 | 79 | 7 | 12 | | 5 | 439 | 10,5 |
| Albertirsa | 7.74 | 526 | 466 | 50 | 24 | 1 | 38 | 4 | 3 | | <1 | 320 | 13,2 |
| Zalaszentgrót | 7.61 | 689 | 627 | 16 | 37 | 3 | 80 | 19 | 105 | | 1 | 329 | 13,5 |
| Somogyvár/1 | 7.40 | 717 | 665 | 38 | 22 | 2 | 64 | 3 | 10 | | 1 | 420 | 17,9 |
| Somogyvár/2 | 7.72 | 722 | 630 | 29 | 39 | 1 | 75 | 13 | 15 | | <1 | 455 | 19,5 |
| Csány | 7.74 | 242 | 611 | 25 | 30 | 1 | 62 | 5 | 5 | | <1 | 428 | 20,9 |
| Zsámbék | 7.56 | 815 | 766 | 15 | 50 | 4 | 112 | 7 | 203 | | <1 | 317 | 38,5 |
| Fonyód | 7.70 | 733 | 741 | 138 | 15 | 2 | 32 | 5 | 10 | | <1 | 535 | 51,8 |
| Kővágóörs | 7.56 | 680 | 610 | 2 | 38 | 5 | 82 | 4 | | 14 | <1 | 451 | 56,3 |
| Mindeszentkála/1 | 6.69 | 1489 | 904 | 33 | 35 | 9 | 140 | 9 | | 145 | <1 | 487 | 125 |
| Pusztazámor | 8.02 | 1034 | 1000 | 258 | 2 | 7 | 6 | 9 | | 110 | <1 | 545 | 194 |
| Detek | 7.75 | 816 | 808 | 205 | 5 | 8 | 8 | 28 | | 1 | <1 | 534 | 203 |
| Mindeszentkála/2 | 7.21 | 985 | 1600 | 38 | 55 | 11 | 272 | 10 | | 35 | <1 | 1100 | 209 |

**Table S4. Mean concentrations (n=10) of selected cations and anions as well as pH, and electric conductivity values determined in Danube River in time period Sept. 2021 – Sept. 2022 at 10 sampling sites**

| **Sampling site** | **River km (km)** | **pH** | **Electric conductivity**  **(µS/cm)** | **Na^+^**  **(mg/L)** | **Mg^2+^**  **(mg/L)** | **K^+^**  **(mg/L)** | **Ca^2+^**  **(mg/L)** | **Cl^-^**  **(mg/L)** | **SO_4_^2-^**  **(mg/L)** | **NO_3_^-^**  **(mg/L)** | **HCO_3_^-^ (mg/L)** |
| --- | --- | --- | --- | --- | --- | --- | --- | --- | --- | --- | --- |
| Medve | 1971 | 7.84 ± 0.19 | 414 ± 44 | 12 ± 2 | 13 ± 1 | 2 ± 0.5 | 50 ± 6 | 17 ± 5 | 24 ± 4 | 6 ± 3 | 202 ± 23 |
| Gönyű | 1805 | 7.84 ± 0.18 | 415 ± 50 | 14 ± 2 | 13 ± 1 | 3 ± 0.8 | 50 ± 7 | 18 ± 5 | 26 ± 4 | 6 ± 3 | 202 ± 18 |
| Szőny | 1761 | 7.89 ± 0.12 | 412 ± 49 | 13 ± 2 | 13 ± 1 | 2 ± 0.5 | 51 ± 6 | 18 ± 5 | 26 ± 5 | 6 ± 3 | 202 ± 22 |
| Dunaalmás | 1752 | 7.72 ± 0.20 | 442 ± 29 | 13 ± 3 | 13 ± 2 | 2 ± 0.6 | 51 ± 6 | 18 ± 6 | 26 ± 5 | 6 ± 3 | 204 ± 20 |
| Göd | 1666 | 7.89 ± 0.15 | 413 ± 47 | 13 ± 2 | 13 ± 1 | 3 ± 0.6 | 49 ± 6 | 18 ± 4 | 26 ± 5 | 6 ± 3 | 200 ± 20 |
| Budapest | 1644 | 7.87 ± 0.17 | 411 ± 62 | 13 ± 3 | 13 ± 1 | 2 ± 0.4 | 51 ± 6 | 17 ± 5 | 26 ± 5 | 6 ± 3 | 205 ± 24 |
| Ercsi | 1613 | 7.86 ± 0.22 | 453 ± 35 | 13 ± 3 | 13 ± 2 | 2 ± 0.5 | 51 ± 6 | 18 ± 5 | 26 ± 5 | 6 ± 3 | 203 ± 19 |
| Dunaföldvár | 1560 | 7.93 ± 0.13 | 469 ± 29 | 14 ± 2 | 14 ± 3 | 3 ± 0.5 | 51 ± 6 | 19 ± 6 | 28 ± 6 | 6 ± 3 | 205 ± 23 |
| Baja | 1480 | 7.97 ± 0.15 | 453 ± 24 | 14 ± 3 | 13 ± 2 | 2 ± 0.5 | 51 ± 3 | 18 ± 6 | 27 ± 5 | 6 ± 3 | 204 ± 23 |
| Mohács | 1446 | 8.02 ± 0.18 | 454 ± 35 | 14 ± 3 | 13 ± 2 | 2 ± 0.6 | 50 ± 6 | 18 ± 6 | 26 ± 5 | 6 ± 2 | 202 ± 21 |

**Figure S1 Temporal changes of Li concentration in the Danube River during the entire sampling period**

**
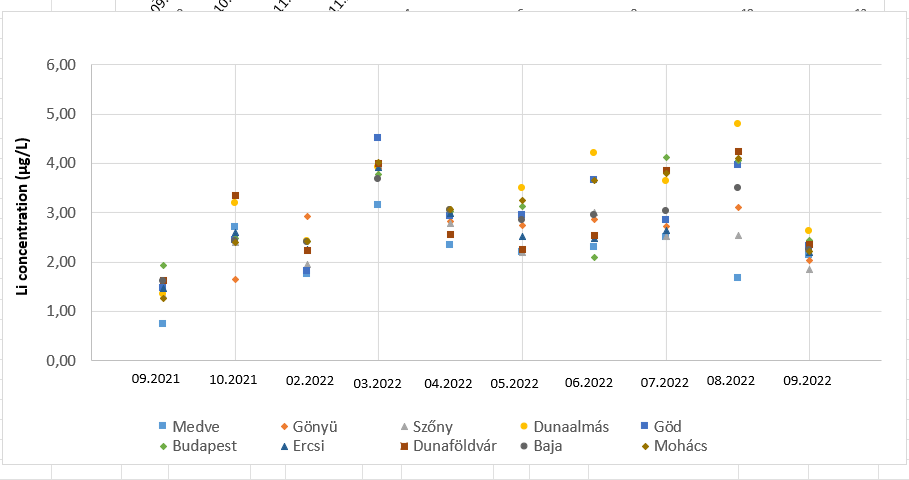
**
